# Promoting Independence in Dementia (PRIDE): A Feasibility Randomized Controlled Trial

**DOI:** 10.2147/CIA.S281139

**Published:** 2021-02-25

**Authors:** Emese Csipke, Aisha Shafayat, Kirsty Sprange, Lucy Bradshaw, Alan A Montgomery, Reuben Ogollah, Esme Moniz-Cook, Martin Orrell

**Affiliations:** 1 Division of Psychiatry, University College London, London, UK; 2 Nottingham Clinical Trials Unit, University of Nottingham, Nottingham, UK; 3 Faculty of Health Sciences University of Hull, Hull, UK; 4 Institute of Mental Health, University of Nottingham, Nottingham, UK

**Keywords:** dementia, feasibility trial, randomized controlled trial, psychosocial intervention

## Abstract

**Background:**

There is a need for interventions to foster and maintain independence for people with dementia to support community living, improve morale, and reduce stigma. We investigated a social intervention to promote living well and enhance independence for people with mild dementia.

**Methods:**

In this two arm parallel group, feasibility RCT at six sites in England, participants were randomized (1:1) to the PRIDE intervention (encompassing social, physical, and cognitive domains supported by a facilitator over three sessions) compared to usual care only. The main objective was to determine the feasibility of a main trial with respect to measures of recruitment, retention, and adherence to the intervention.

**Results:**

During a 7-month period, 402 people were invited to the trial, 148 were screened (37%, 95% confidence interval (CI)=32–42%), 137 were eligible at pre-consent, 94 consented to the trial (69% of those eligible, 95% CI=60–76%), and 92 were randomized (46 to each group). Of those allocated to the intervention, 42 (91%) received at least one of three intervention sessions. Outcome assessment follow-up visits were completed for 73 participants at 6 months (79%, 95% CI=70–87%), and this was similar for both groups.

**Conclusion:**

A large multi-center trial of the PRIDE intervention in community-dwelling people with mild dementia is feasible using systematic recruitment strategies. The intervention was successfully delivered and well received by participants. Findings from this study will be used to refine the design and processes for a definitive RCT.

**Trial Registration:**

ISRCTN, ISRCTN11288961, registered on 23 October 2018.

## Background

People with dementia lose independence for many reasons, such as neurological deterioration, reduced living skills, or negative social consequences, such as stigma, social exclusion, and disempowerment. Receiving specialist support soon after diagnosis may facilitate independence in early stage dementia. Family and friends may also inadvertently contribute to a reduced sense of autonomy in people with dementia by a lowered set of expectations, and well intentioned “taking over” of decision-making and responsibilities in order to support their relative.^1^ Fundamental concerns for people with dementia include loss of power in social relationships, the need to maintain active roles outside their immediate social networks, and a dearth of information on diagnosis, prognosis, and post-diagnostic support services. Both the UK government and the EU Joint Program for Neurodegenerative Disease Research have underscored the need for high quality psychosocial interventions, to support the growing post-diagnostic needs of people with dementia.^2^

The Promoting Independence in Dementia (PRIDE) program was developed as a post-diagnostic social intervention^3^,^4^ to support independence and quality-of-life for people with early stage dementia, so that they could live well and as independently as possible in the community. The PRIDE intervention includes the principles of self-management as applied to the treatment of chronic conditions,^5^ including teaching the individual how to manage their condition and identify solutions specific to their needs.^6^ Strategies can include decision-making, identifying and using available resources, problem-solving, and being an active participant in choices about care, in partnership with healthcare professionals.^7^,^8^ PRIDE promotes social inclusion, harnesses the support of the person’s social network, and facilitates engagement in stimulating cognitive, physical, and social activities. The intervention is aimed at those with mild dementia who are likely to have minor difficulties with daily living activities. It was manualized using the conceptual frameworks and associated mechanisms of action that were identified within the research program.^9^ It allows individualized tailoring according to the person’s needs and circumstances using co-production approaches to develop the intervention.

Previous groundwork involved a mixed-methods non-randomized, pre–post feasibility study, conducted at four sites.^10^ This found that the intervention was acceptable where 73% completed all three sessions. However, recruitment and delivery within the voluntary sector was not effective. The present feasibility study was delivered in an alternative setting, utilizing NHS services for delivery of the intervention. We also wished to examine whether the variable recruitment rates we noted previously could be improved using site-specific requirements. This information about availability of intervention facilitators and site-based requirements for recruitment is of key importance for determining the feasibility of a future large-scale definitive RCT.

The aim of this study was to investigate the feasibility of the PRIDE intervention, within a randomized controlled design to inform a future large scale definitive RCT of the clinical and cost effectiveness. This study examined in detail screening, recruitment, and follow-up rates, and the potential of clinical outcome measures within a RCT design, as we were interested in completion rates of follow-up data, particularly for those not allocated to the intervention group. The embedded qualitative study, fidelity assessment, and data to inform the cost-effectiveness analysis will be reported elsewhere.

## Methods/Design

This PRIDE randomized feasibility study was designed as a two arm parallel group multi-center study with participants individually allocated on a 1:1 ratio to usual care or usual care plus the PRIDE intervention. It also included an embedded qualitative process evaluation (see protocol^11^ for design and methods).

### Participants

Recruitment took place in six NHS sites across England, with participants identified via the NHS, Joint Dementia Research (JDR; an online research register) and self-referral. Participants were eligible if they were within the selected site-catchment area; aged over 18 years; met the Diagnostic and Statistical Manual of Mental Disorders-Fourth Edition criteria for dementia of any type; were able to provide informed consent and engage in the intervention in the opinion of the investigator (or designee); were able to read/communicate in English; and were not living in institutional care. In addition, the participant must have had mild dementia, defined as a score of 0.5 or 1 on the Clinical Dementia Rating (CDR) Scale, which was assessed at the baseline visit after consent. Participants were able to take part with or without a supportive other (eg, carer). If taking part, the supporter was eligible if they were aged 18 or over; able to engage with and participate in the intervention; and able to provide informed consent and read and communicate verbally in English. After identification, a member of the site research team contacted the potential participant to confirm initial eligibility and to arrange a baseline assessment. At the home visit, the researcher explained the study, obtained written informed consent, and collected baseline data including the CDR scale^12^ to confirm eligibility.

Follow-up visits were conducted at 3 and 6 months post-randomization in the participant’s home by a site research team member blind to group allocation. Questionnaires for supporters were either completed at the visit if the supporter was present or left with a prepaid envelope for the supporter to return to the coordinating center.

### Facilitation

#### Training

Training by the research team involved a 1-day session developed through co-production and consultation with voluntary sector dementia advisors and NHS memory clinic nurses. The theories underlying the intervention were provided, followed by group work, role play, and discussion. Facilitators all had a background of working with dementia. Trainers were available throughout the study for continued support.

##### Usual Care and Intervention

All participants received the services usually available to people with dementia at the participating sites. In addition, participants allocated to the intervention arm received the PRIDE intervention.^4^ The three-session intervention is delivered by trained intervention facilitators. Each session lasts between 1–2 hours conducted in a venue selected by the participant, usually their own home. Working together, the intervention provider and participant develop a personalized profile for the person with dementia. This is followed by a collaborative approach to planning for doing activities that are important to the person with dementia. These plans are reviewed in subsequent sessions and modified if necessary. A paper manual guides the intervention and includes signposting to information and resources, and an electronic version was also available allowing participants to choose either or both if they wished.^4^

### Outcomes

#### Feasibility Outcomes

The primary outcome of this study was the feasibility set within an RCT design to explore further what is required for delivery of large scale definitive RCT of the PRIDE intervention. The objectives and outcomes are shown in Table 1.

**Table 1:** Feasibility Objectives and Outcomes

Feasibility Objectives	Feasibility Outcomes
1. Determine the feasibility of recruitment to a large-scale RCT	Aggregate data on potential participants within NHS services Number of patients assessed for eligibility/consented/randomized Number and proportion of potential participants identified through NHS services, Join Dementia Research, and by self-referral who are eligible Reasons for non-inclusion/non-eligibility Monthly recruitment rate per site Barriers and facilitators to recruitment (Interviews/focus groups)
2. Refine the eligibility criteria for a future definitive RCT	Number of screening failures for eligibility, post-consent Participant and facilitator report (Interviews/focus groups)
3. Determine the acceptability to patients/clinicians of randomization	Proportion of eligible patients that consent to randomization Reasons for non-consent Participant and facilitator report (Interviews/focus groups)
4. Determine the relevance and acceptability to patients/clinicians of the trial intervention	Premature discontinuation or non-attendance of treatment and reasons Feedback from participants and site staff delivering the intervention Participant and facilitator report (Interviews/focus groups)
5. Determine the acceptability to patients/clinicians of the trial procedures	Proportion of approached NHS sites that agree to participate in the trial and reasons for non-participation Proportion of eligible patients that consent to randomization Reasons for non-consent Withdrawals and losses to follow-up and reasons Feedback from participants and staff (Interviews/focus groups)
6. Assess the ability of NHS sites to deliver the intervention	Measures of the feasibility of delivering the PRIDE intervention within NHS settings: number/grade/experience of staff within the service, staff turnover, and time to treatment initiation Measures of the recruitment and retention of PRIDE facilitators during the study treatment period Barriers to treatment delivery per protocol (Interviews/focus groups)
7. Assess training and support needs for NHS staff delivering the intervention	Feedback on training delivered (Interviews/focus groups) Support offered/accepted (eg, log of calls and emails to central support lines)
8. Evaluate treatment fidelity when delivered through NHS services	Measures of treatment fidelity including: adherence to intervention manual and uptake of activities Feedback from participants and staff (Interviews/focus groups)
9. Determine the services and interventions provided as usual care and evaluate methods for measuring this	Post-diagnostic care pathway Services available Uptake of services
10. Assess follow-up and outcome completion rates	Response rate to follow-up assessment Questionnaire completion rates Amount of missing questionnaire data at item and scale levels
11. Determine the relevance and acceptability of a range of clinical outcome measures and selection of the primary outcome for the main trial	Completion rates and reasons for non-completion/missing data Estimates of clinically important differences, variance, and sensitivity to change for the clinical outcome measures Direct questions to participants regarding relevance of measures
12. Evaluate the utility and acceptability of resource use questionnaires for use in an economic evaluation alongside a future RCT	Completion rate and reasons for non-completion/missing data
13. Comparative micro-costing of PRIDE intervention and usual care	Staff time and resources for delivery of PRIDE intervention Other service use
14. Estimate the sample size required for a definitive study	Primary outcome selection Variability in the outcome Withdrawals and losses to follow-up
15. Determine the resources required for a full trial	Sample size, recruitment rate (number of sites/recruitment period), staffing and resources (for recruitment, treatment and follow-up)

#### Clinical Outcomes

Nine clinical outcomes measures for participants, described in Table 2 to collect data on domains recommended to evaluate psychosocial interventions in dementia,^13^,^14^ were collected at the baseline, 3, and 6 months. These outcomes covered Activities of Daily Living,^15^ Quality-of-Life (DEMQoL^16^), EQ5D-5L,^17^, Mood (GDS-15^18^), Cognition (SMMSE^19^), Wellbeing (CASP^20^), Quality of relationships (IPAQ-O^21^), and Positive emotions (PPOM^22^). In addition, participants were asked to provide a rating of their perceived change in wellbeing and independence compared to the start of the study (global change) to be used to explore the responsiveness to change of the clinical outcome measures. Supporters were similarly asked to provide a rating of their perceived change for the person with dementia in wellbeing and independence. Questionnaires were read out to participants to ensure consistency and promote inclusiveness for those who found reading text difficult.

**Table 2:** Summary of the Clinical Outcome Measures

Outcome Measures	Scale, Description, and Source	Derivation of Scores^A^
**Participants**		
**Activities of Daily Living**	Measured using the Lawton Instrumental Activities of Daily Living (IADL) Scale^15^ Performance is measured across eight domains	Each domain has between three and five response options describing ability. These are scored as 0 (less able) or 1 (more able) Domain scores summed to produce summary score from 0 (low function) to 8 (high function)
**Health-related quality-of-life**	Measured using EuroQoL Quality-of-Life Questionnaire – 5 Domains, 5 Levels^17^ Consists of two parts: a descriptive system and a visual analog scale (VAS) asking about health on that day The 5 level descriptive system used as part of the health economic analysis and VAS summarized in the quantitative analysis of the clinical outcomes	VAS scores range from 0 (worst health can imagine) to 100 (best health can imagine)
**Quality-of-life**	Measured using DEMQoL^16^ Assesses five domains of quality-of-life including health and well-being, cognitive functioning, social relationships and self-concept	Scale consists of 28 items about the last week with four response options (a lot, quite a bit, a little, and not at all) Items are scored as 1=a lot, 2=quite a bit, 3=a little, and 4=not at all, apart from five positive questions, which are scored in reverse Items are summed to produce a total score ranging from 28–112, higher scores indicating better quality-of-life Missing items are imputed with person-specific mean of completed items provided at least 50% of items are complete (ie, 14 items)
**Mood**	Measured using the Geriatric Depression Scale (GDS) – short form^18^	15-items about the last week with responses of yes or no Items scored as 1 when response indicates depression and 0 otherwise (10 items indicate the presence of depression when answered positively, and five items when answered negatively) Item scores summed to produce a total score ranging from 0–15, with higher scores indicating more severe depression
**Cognition**	Measured using the Standardized Mini Mental State Exam (S-MMSE)^19^	Brief assessment of cognition testing orientation (time and place), repetition, verbal recall, attention and calculation, language and visual construction The total test score is the sum of the correct responses ranging from 0–30, with a lower score indicating more cognitive impairment An adjusted score can be calculated for people who are physically unable to do some of the tasks on the MMSE (IHPA Australia)
**Wellbeing**	Control, Autonomy, Self-realization, and Pleasure (CASP) questionnaire^20^ Assesses quality-of-life in older people across four domains; control, autonomy, pleasure, and self-realization	19 items with four response options (often, sometimes, not often, and never) Items scored on a 4-point Likert scale as shown on the CASP website (https://casp19.com/casp-scoring-and-properties/). Items scores summed to produce a total score ranging from 0–57, with higher scores indicating better quality-of-life. The 12 item version of the CASP also used as this has been found to have a better factor structure for people with dementia than the 19 item.^19^ In the 12 item version, items 3 (control subscale), items 6 and 8 (autonomy subscale), items 13 and 14 (pleasure subscale), and items 16 and 17 (self-realization subscale) are not used
**Quality of relationships**	Measured using Impact on Participation and Autonomy Questionnaire for older people (IPAQ-O) social relations subscale^21^	Social relations subscale has five items with five response items The response options on the PRIDE CRF were very good, good, fair, poor, and very poor from the original IPAQ^23^ rather than amended response options for the IPAQ-O (totally agree, partly agree, neither agree nor disagree, disagree, and totally disagree) The response options were scored as on the original IPAQ: very good=1, good=2, fair=3, poor=4, and very poor=5 Item scores summed to produce a total score ranging from 5–25, with higher score indicating more restriction in participation
**Positive emotions**	Measured using the Positive Psychology Outcome Measure (PPOM)^22^ Two subscales: hope and resilience	16-item scale measuring aspects of positive psychology with an eight item hope subscale and an eight item resilience subscale Each item is rated on a 5-point Likert scale (0=not true at all, 1=rarely true, 2=sometimes true, 3=often true, 4=true nearly all the time) Items scores summed to produce a total score ranging from 0–64 and subscale scores ranging from 0–32, higher scores indicate better wellbeing
**Social engagement**	Measured using the number of social contacts and leisure activities in the preceding 2 weeks	Questions asked: How many times over the last 2 weeks have you met up with friends or family? (eg, family or friends visiting, you visiting them, attending a social club) How many times over the last 2 weeks have you participated in any leisure activities? (eg, hobbies)
**Global change in wellbeing and independence at 3 and 6 months**	Participants were asked to provide a rating of their perceived change using a 5-point ordinal scale.	Questions asked: Compared to 3/6 [depending on time since randomization] months ago when you started in the PRIDE study, how would you rate your general wellbeing now? Response options: much better, a bit better, no change, a bit worse, much worse Compared to 3/6 [depending on time since randomization] months ago when you started in the PRIDE study, how independent do you feel now? Response options: much more independent, a bit more independent, no change, a bit less independent, much more independent
	Supporters were asked to independently provide a rating of their perceived change for the person with dementia from their perspective, during the course of the study, using the same 5-point ordinal scale	Questions asked: Compared to 3/6 months ago [depending on time since randomization] when your friend/relative started in the study, how would you rate their general wellbeing now? Compared to 3/6 months ago [depending on time since randomization] when your friend/relative started in the study, how independent do you feel they are now?
**Supporters^B^**		
**Health-related quality-of-life**	Measured using the EuroQoL Quality-of-Life Questionnaire – 5 Domains, 5 Levels Consists of two parts: a descriptive system and VAS The 5 level descriptive system used as part of the health economic analysis and VAS summarized in the quantitative analysis of the clinical outcomes	VAS scores range from 0 (worst health can imagine) to 100 (best health can imagine)

**Notes:** ^A^For missing data within questionnaires, tool-specific guidance was used to derive scores if available. Otherwise missing items were imputed by the participant mean of the completed responses (rounded to one decimal place) in order to derive scores.^24^ BMood measured using the General Health Questionnaire was listed as an outcome for supporters in version 1.0 (Sept 18, 2018) of the protocol, however this was removed in version 2.0 (Dec 20, 2018) to reduce the burden on participant and supporters in completing questionnaires and was not collected for any supporters.

### Sample Size

As this was a feasibility study, a formal sample size calculation for between group comparisons of a primary outcome was not appropriate. A target sample size of 75–80 participants was set over the recruitment period to establish recruitment capability of the range of participating services. Seventy-five participants at randomization allowed estimation of recruitment with a margin of error (half-width of 95% confidence interval) of around 8 percentage points, and retention of 12 percentage points.

### Randomization

Participants were allocated at the individual level to intervention or control on a 1:1 ratio using a minimization algorithm with a probabilistic element, created by the Nottingham Clinical Trials Unit (NCTU). The minimization variables were site, gender, age (<80 or ≥80) and medication for dementia (any vs none). The investigator or “authorized designee” randomized participants, following completion of the baseline assessments, using a remote, internet-based randomization system. Following randomization, participants were notified of their treatment allocation by an unblinded member of the research team. Researchers remained blind to allocation.

### Statistical Analyses

Data analysis were primarily descriptive. All analyses were documented in a Statistical Analysis Plan which was finalized prior to database lock. Feasibility outcomes were estimated using descriptive statistics (with 95% confidence intervals – CI – if relevant) and included recruitment rates, follow-up rates in both arms of the trial, missing data, and intervention adherence. Demographic and clinical characteristics at baseline were summarized in the two allocated groups. Clinical outcomes were summarized descriptively for participants with outcome data regardless of adherence with the allocated intervention.

Three clinical outcomes, the Lawton Instrumental Activities of Daily Living (IADL) scale, quality-of-life using the DEMQoL and wellbeing using Control, Autonomy, Self-realization, and Pleasure scale (CASP, scored from both 19-item and 12-item version), were chosen as candidate primary outcomes to evaluate their responsiveness and minimal important change (MIC);^25^ against the global change questions at 6 months (see Supplementary Methodology 1).

It was not an objective of this feasibility study to obtain definitive estimates of the intervention effect on clinical outcomes as it was not powered to do so. However, differences in means between groups (with CIs) for the candidate primary outcomes described above at 6 months were calculated using linear mixed models, adjusted for the minimization variables with a random effect for the recruiting site, to show the possible range of treatment effects. Adjusted differences in means are presented with 95%, 85%, and 75% CIs in forest plots to explore the strength of the preliminary evidence.^26^

## Results

### Sites and Recruitment

Fifty-three sites were approached through a national Memory Services register, of which 29 (55%) expressed an interest and 19 (36%) returned the study eligibility questionnaire. Of these, 12 (63%) met the eligibility criteria and six were selected. Sites were geographically spread and covered urban and rural areas, a wide socio-economic distribution, and varied in terms of the size of the populations they served. Overall, 46 research staff were involved in the study, ranging from three to 12 per site. Nineteen staff were trained to be facilitators, and comprised of nurses, occupational therapists, clinical researchers, and assistant psychologists.

**Figure 1:**
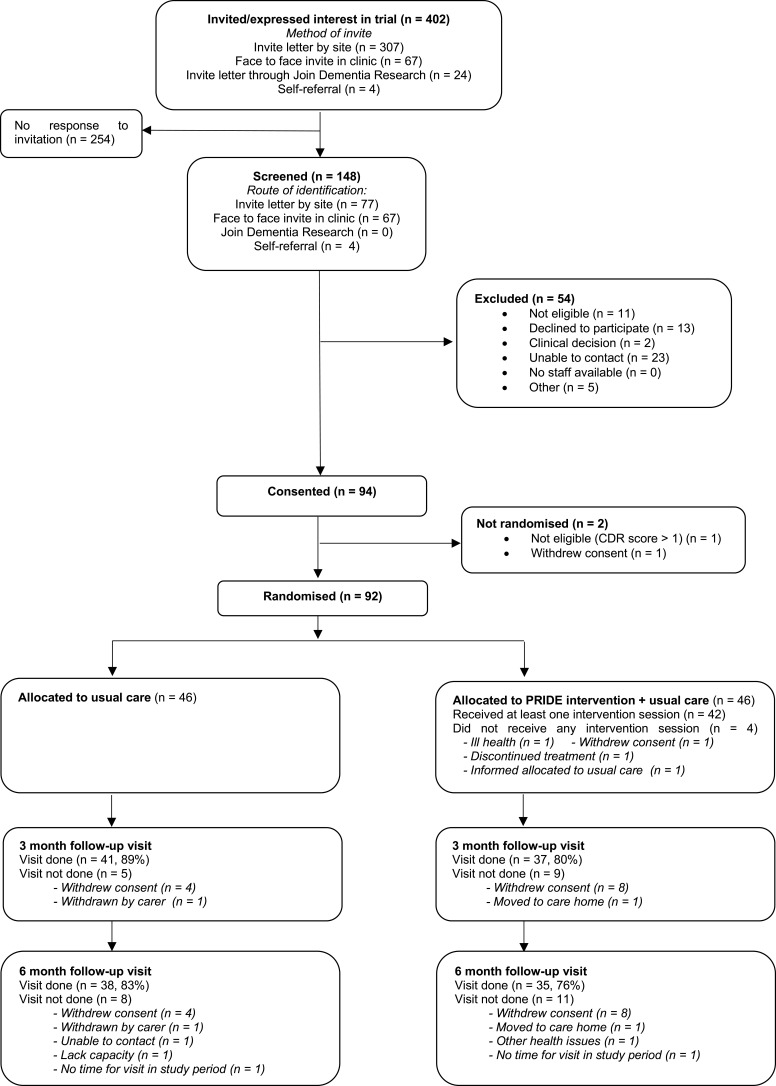
Participant flow diagram.

The sites opened to recruitment between November 2018 and February 2019. Figure 1 summarizes the participant flow into the trial. Of the 402 people invited, 148 were screened (37%, 95% CI=32–42%), with a mean age of 77 years (SD=8.1) and half were female. Of those screened, 137 (93%) were eligible pre-consent, 94 consented to the trial (69% of those eligible, 95% CI=60–76%), and 92 were randomized (67%, 95% CI=59–75%), 46 to each arm. Following screening, one site contributed to most of the 54 “non-consenting” participants and, of those, the majority could not be contacted again after the initial approach (n=23) or declined to participate (n=10). The number of participants randomized per site ranged from 11–22, with a mean of 2.6 participants randomized per month.

Randomized participants had a mean age of 78 (SD=8.0), half were female, most were of white ethnicity (93%), and approximately two thirds had Alzheimer’s type dementia (Table 3). Baseline characteristics were in general well balanced across the two groups, although slightly more participants in the PRIDE intervention group were married (or had a partner) and lived with others compared to the usual care group (Table 3). Around two thirds of participants chose to take part with a supporter and the majority of supporters were a spouse or partner (Table 3).

**Table 3:** Participant Baseline Characteristics Data

	Usual Care (n=46)	PRIDE Intervention (n=46)	Total (n=92)
Age at randomization (years)			
Mean [SD]	78.6 [7.1]	77.3 [8.9]	78.0 [8.0]
Median [25th, 75th centile]	79 [74, 85]	78 [72, 84]	78 [73, 84]
Min, max	56, 90	51, 93	51, 93
≥80	21 (46%)	20 (43%)	41 (45%)
Gender			
Male	23 (50%)	23 (50%)	46 (50%)
Female	23 (50%)	23 (50%)	46 (50%)
Ethnicity			
White	42 (91%)	44 (96%)	86 (93%)
Black	1 (2%)	–	1 (1%)
Asian	2 (4%)	2 (4%)	4 (4%)
Mixed	1 (2%)	–	1 (1%)
Educational attainment			
Primary	–	–	–
Secondary	31 (67%)	26 (57%)	57 (62%)
Higher	15 (33%)	19 (41%)	34 (37%)
Not known	–	1 (2%)	1 (1%)
Living arrangements			
Lives alone	15 (33%)	9 (20%)	24 (26%)
Lives with others	31 (67%)	37 (80%)	68 (74%)
Marital status			
Single/divorced/widowed/separated	16 (35%)	10 (22%)	26 (28%)
Married/with partner	30 (65%)	36 (78%)	66 (72%)
Facilities available for use of web-based manual (ie, computer and internet access)	17 (37%)	16 (35%)	33 (36%)
Type of dementia			
Alzheimer’s Type	31 (67%)	28 (61%)	59 (64%)
Vascular	10 (22%)	9 (20%)	19 (21%)
Lewy body	–	2 (4%)	2 (2%)
Mixed	3 (7%)	5 (11%)	8 (9%)
Not known	1 (2%)	1 (2%)	2 (2%)
Other (frontotemporal dementia)	1 (2%)	1 (2%)	2 (2%)
Medication taken for dementia^A^	32 (70%)	32 (70%)	64 (70%)
Clinical Dementia Rating Scale score			
0.5 (very mild)	28 (61%)	35 (76%)	63 (68%)
1 (mild)	18 (39%)	11 (24%)	29 (32%)
Supporter consented to participate	34 (74%)	30 (65%)	64 (70%)
Spouse/partner	23	24	47
Son/daughter	9	5	14
Friend or another relative	2	1	3

**Notes:** Data are N (%) unless otherwise indicated. ^A^Medication for dementia include any of the following: Donepezil Hydrochloride, Rivastigmine, Memantine Hydrochloride, and Galantamine.

Of those allocated to the intervention, 42 (91%) received at least one intervention session, with 42 (91%) attending the first session, 34 (74%) the second, and 33 (72%) the third session. The main reason for non-attendance at sessions was withdrawal from the study, occurring at session 2 (n=5) and session 3 (n=6). The paper manual was used for all participants, apart from one participant who used both the paper and electronic versions.

Outcome assessment visits were completed for 78 participants (85%, 95% CI=76–91%) at 3 months and 73 (79%, 95% CI=70–87%) at 6 months and completion was similar in the two groups (Figure 1). Outcome assessment visits were not completed due to withdrawal of consent from the trial and at 6 months due to being unable to organize a visit within the time frame (Figure 1). Participants who did not complete the visit at 6 months were slightly older than those completing the visit (mean=80 years [SD=7] vs mean 77 years [SD=8]). At the visits, completion of the clinical outcomes was high. All assessments were completed sufficiently to derive a score apart from the standardized mini mental state exam (sMMSE) which was not fully completed by two participants at 3 months and four participants at 6 months, one participant refused to do the PPOM at 6 months and there was insufficient time for completion of the sMMSE and CASP at the 6 month visit for one participant. Between 80% and 90% of participants at each time point agreed or strongly agreed that the questions in the EQ-5D and the DEMQoL were relevant to them with between 70% and 80% agreeing or strongly agreeing for the other measures. Of the 64 supporters who consented to the trial, 55 (86%) completed the follow-up questionnaire at 3 months and 52 (81%) at 6 months.

At 6 months, 36% of participants (26/73) rated their wellbeing as a bit better or much better compared to when they started in the study and 22% (16/73) responded that they were a bit more or much more independent (Table 4). Supporters responded that participant wellbeing was a bit/much better for 13% (7/52) and that the participant was a bit/much more independent for 11% (6/52). The area under the ROC curves (AUC) for improvement in wellbeing using the participant rating at 6 months according to change in baseline in the candidate primary outcomes ranged from 0.55–0.74 (Table 5) with the greatest area observed for the CASP12. Similar values were observed for the AUC using the participant rating of independence as the external criterion for improvement. The AUC for no decline in independence using the global change question was also evaluated in a post hoc analysis with change in CASP12 having the greatest AUC for no decline (0.65, 95% CI=0.48–0.83). All estimates of the minimal important change for the candidate primary outcome measures shown in Table 6 have wide confidence intervals, although estimates in general were fairly similar using either the participant or supporter rating of change for each estimation method.

**Table 4:** Wellbeing and Independence: Participant Rating of Change in General Well-Being and Independence at 6 Months Compared to When They Started in the PRIDE Study (Global Change)

	Usual Care	PRIDE Intervention	Total
Questionnaire completed at 6 months	38	35	73
General wellbeing			
Much worse	–	–	–
A bit worse	7 (18%)	6 (17%)	13 (18%)
No change	18 (47%)	16 (46%)	34 (47%)
A bit better	8 (21%)	8 (23%)	16 (22%)
Much better	5 (13%)	5 (14%)	10 (14%)
Independence			
Much less independent	2 (5%)	–	2 (3%)
A bit less independent	8 (21%)	5 (14%)	13 (18%)
No change	20 (53%)	22 (63%)	42 (58%)
A bit more independent	6 (16%)	4 (11%)	10 (14%)
Much more independent	2 (5%)	4 (11%)	6 (8%)

**Note:** Percentages use the number of participants completing the questionnaire at 6 months as the denominator.

**Table 5:** Responsiveness to Change at 6 Months for Candidate Primary Outcome Measures

	IADL		DEMQOL		CASP19		CASP12	
	Participant (n=73)	Supporter (n=52)	Participant (n=73)	Supporter (n=52)	Participant (n=72)	Supporter (n=52)	Participant (n=72)	Supporter (n=52)
**Well-being**								
Spearman correlation between change from baseline at 6 months and global change in wellbeing question	0.19	0.26	0.08	0.22	0.34	0.33	0.40	0.24
Area under the ROC curve for improvers in wellbeing according to change from baseline at 6 months (95% CI)	0.61 (0.48–0.74)	0.59 (0.36–0.83)	0.55 (0.41–0.69)	0.73 (0.53–0.94)	0.71 (0.59–0.83)	0.66 (0.45– 0.87)	0.74 (0.62–0.85)	0.59 (0.37–0.81)
**Independence**								
Spearman correlation between change from baseline at 6 months and global change in independence question	0.17	0.09	−0.08	0.29	0.29	0.49	0.34	0.41
Area under the ROC curve for improvers in independence according to change from baseline at 6 months (95% CI)	0.67 (0.50–0.84)	0.54 (0.29–0.79)	0.45 (0.28–0.61)	0.68 (0.42–0.95)	0.70 (0.57–0.83)	0.72 (0.52–0.93)	0.73 (0.60–0.85)	0.74 (0.50–0.97)

**Table 6:** Estimates of the Minimal Important Change (MIC) at 6 Months for the Candidate Primary Outcomes Measures

	Using Participant Response to Global Change Question MIC Estimate (95% CI)	Using Supporter Response to Global Change Question MIC Estimate (95% CI)
**Using global change in well-being as anchor**		
IADL		
Between patient score change	0.2 (−0.6 to 1.0)	−0.2 (−1.6 to 1.1)
Sensitivity/specificity approach	0.4 (−2 to 2)	0 (−2 to 2)
DEMQoL^A^		
Between patient score change	3.6 (−3.1 to 10.3)	4.6 (−6.7 to 15.8)
Sensitivity/specificity approach	5 (−7 to 18)	12 (0 to 14)
CASP19		
Between patient score change	3.5 (0.1 to 6.9)	3.7 (−4.5 to 11.8)
Sensitivity/specificity approach	−3 (−3 to 3)	1 (−3 to 7)
CASP12		
Between patient score change	2.3 (−0.1 to 4.7)	2 (−4 to 7.9)
Sensitivity/specificity approach	1 (−3 to 3)	−3 (−3 to 5)
**Using global change in independence as anchor**		
IADL		
Between patient score change	0.6 (−0.4 to 1.6)	−0.6 (−2 to 0.8)
Sensitivity/specificity approach	0.4 (−1 to 2)	0 (−2 to 2)
DEMQoL^A^		
Between patient score change	Not calculated^A^	1.7 (−8.7 to 12.1)
Sensitivity/specificity approach		6 (−4 to 18)
CASP19		
Between patient score change	2.6 (−1.7 to 6.8)	2.6 (−3.8 to 8.9)
Sensitivity/specificity approach	0 (−1 to 2)	1 (−2 to 8)
CASP12		
Between patient score change	1.6 (−1.4 to 4.7)	1.3 (−3.1 to 5.7)
Sensitivity/specificity approach	0 (−1 to 3)	4 (−3 to 5)

**Notes:** ^A^MIC not calculated for the DEMQoL using the participant rating of independence at 6 months: For the between patient score change approach as the mean change in DEMQoL score for participants rating themselves as a bit more independent was less than the mean change in the group rating themselves as having no change in independence. For the sensitivity/specificity approach as Table 5 shows that discriminatory ability of DEMQoL to discriminate between improvers and non-improvers is less than chance (area under curve <0.5). Between patient score change: MIC calculated as the difference between the mean change in the group with a response of a bit better/a bit more independent and the group with a response of no change on the global change questions. Sensitivity/specificity approach: MIC defined as the change from baseline that maximizes the Youden Index (sensitivity + specificity – 1) to discriminate between improvers and non-improvers. Confidence intervals for sensitivity/specificity approach estimated using bootstrapping with 1,000 repetitions. IADL scores range from 0 (low function) to 8 (high function). DEMQoL scores range from 28– 112, higher scores indicating better quality-of-life. CASP scores for 19 item version range from 0–57 and for 12 item version range from 0–36, with higher scores indicating better quality-of-life.

**Abbreviations:** IADL, Lawton Instrumental Activities of Daily Living Score; CASP, Control, Autonomy, Self-realization, and Pleasure Scale.

**Figure 2:**
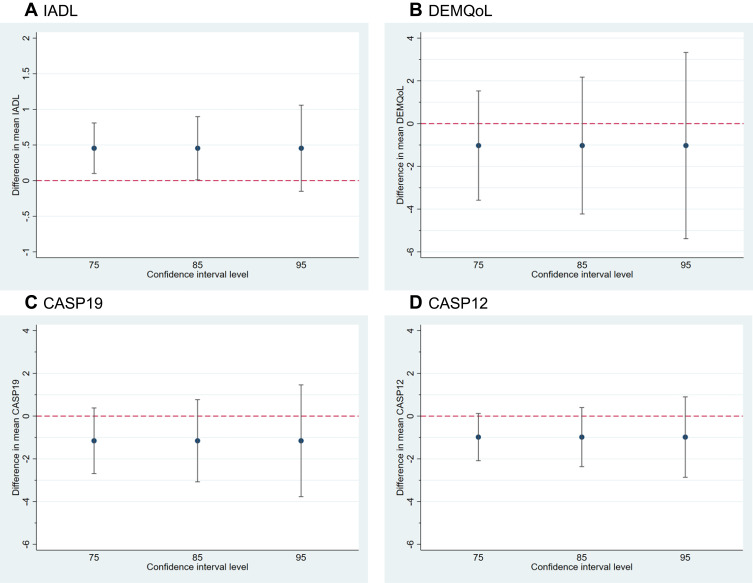
Difference in means (intervention – control) at 6 months with confidence intervals for the candidate primary outcomes.

**Notes:** Positive values indicate improvement for all outcomes. (**A**) Difference in mean Instrumental Activities of Daily Living at 6 months. (**B**) Difference in mean DEMQoL (quality-of-life) at 6 months. (**C**) Difference in mean Control, Autonomy, Self-realization, and Pleasure scale 19 item version at 6 months. (**D**) Difference in mean Control, Autonomy, Self-realization, and Pleasure scale 12 item version at 6 months.

Scores on the clinical outcomes were similar in the two groups at all time points (Table 7). In Figure 2, 95% confidence intervals for the difference between groups include 0 for all of the candidate primary outcome measures. The 85% and 75% CIs are supportive of small differences favoring the intervention group for the IADL, however in contrast for the CASP 85% and 75% CIs mostly lie below 0 (ie, favoring the usual care group) with upper limits between 0 and 1.

**Table 7:** Participant Clinical Outcomes Summary by Allocated Group

	Baseline Mean [SD]	3 Months Mean [SD]	6 Months Mean [SD]
Lawton Instrumental Activities of Daily Living total score			
Usual care	5.6 [1.5] (n=46)	5.2 [1.7] (n=41)	4.7 [1.7] (n=38)
PRIDE intervention	5.0 [1.7] (n=46)	4.6 [2.1] (n=37)	4.8 [2.1] (n=35)
EQ-5D-5L health status VAS score			
Usual care	73.0 [19.5] (n=45)	73.8 [15.6] (n=41)	75.1 [17.4] (n=38)
PRIDE intervention	70.5 [17.2] (n=46)	68.4 [18.7] (n=37)	67.4 [17.9] (n=34)
DEMQoL total score			
Usual care	88.0 [14.3] (n=46)	90.3 [14.1] (n=41)	91.0 [15.0] (n=38)
PRIDE intervention	87.8 [13.5] (n=46)	90.1 [13.7] (n=37)	89.5 [12.7] (n=35)
Geriatric Depression Scale total score			
Usual care	3.8 [3.5] (n=46)	3.4 [3.1] (n=41)	3.4 [3.0] (n=38)
PRIDE intervention	4.6 [3.5] (n=46)	4.1 [2.8] (n=37)	3.8 [3.3] (n=35)
Standardized Mini Mental State Exam total score			
Usual care	23.6 [3.6] (n=45)	23.7 [3.2] (n=40)	23.4 [3.9] (n=35)
PRIDE intervention	24.3 [4.0] (n=46)	24.4 [3.4] (n=36)	24.2 [4.3] (n=33)
Control, Autonomy, Self-realization, and Pleasure Scale (CASP-19)			
Usual care	41.3 [8.6] (n=46)	42.0 [8.3] (n=41)	42.5 [8.8] (n=37)
PRIDE intervention	39.1 [9.7] (n=46)	40.7 [8.9] (n=37)	39.4 [10.1] (n=35)
CASP-12			
Usual care	25.8 [5.8] (n=46)	26.5 [6.0] (n=41)	26.7 [6.1] (n=37)
PRIDE intervention	24.6 [6.7] (n=46)	25.8 [6.0] (n=37)	24.6 [7.0] (n=35)
Impact on Participation and Autonomy (IPAQ-O) – Social Relations Sub-Scale^A^			
Usual care	9.4 [2.9] (n=46)	9.6 [2.4] (n=41)	8.8 [2.4] (n=38)
PRIDE intervention	9.2 [3.2] (n=46)	8.7 [2.6] (n=37)	8.5 [2.7] (n=35)
Positive Psychology Outcome Measure (PPOM)			
Total			
Usual care	47.9 [9.8] (n=46)	47.5 [9.6] (n=41)	47.4 [10.4] (n=38)
PRIDE intervention	46.6 [10.8] (= 46)	46.3 [10.3] (n=37)	47.6 [7.8] (n=34)
Hope			
Usual care	24.4 [5.1] (n=46)	24.9 [4.8] (n=41)	24.3 [5.3] (n=38)
PRIDE intervention	24.3 [5.5] (n=46)	23.8 [5.7] (n=37)	24.3 [3.9] (n=34)
Resilience			
Usual care	23.5 [5.4] (n=46)	22.7 [5.6] (n=41)	23.1 [5.8] (n=38)
PRIDE intervention	22.3 [6.0] (n=46)	22.5 [5.3] (n=37)	23.2 [5.5] (n=34)
Social engagement			
Number of times in the last 12 weeks met up with friends or family			
Usual care	6.9 [5.8] (n=46)	7.7 [5.2] (n=40)	7.4 [4.6] (n=38)
PRIDE intervention	6.3 [4.6] (n=46)	8.3 [6.1] (n=37)	8.1 [6.7] (n=35)
Number of times over the last 12 weeks participated in any leisure activities			
Usual care	5.5 [5.2] (n=45)	6.7 [5.4] (n=41)	4.6 [4.4] (n=38)
PRIDE intervention	8.7 [12.0] (n=46)	8.6 [6.9] (n=36)	7.2 [7.0] (n=34)

**Notes:** ^A^The response options on the PRIDE CRF for the IPAQ-O were very good, good, fair, poor, and very poor from the original IPAQ rather than amended response options for the IPAQ-O (totally agree, partly agree, neither agree nor disagree, disagree, and totally disagree). Items scored as on the original IPAQ to derive the IPAQ-O social relations score. IADL scores range from 0 (low function) to 8 (high function). EQ-5D-5L VAS scores range from 0 (worst health can imagine) to 100 (best health can imagine). DEMQoL scores range from 28–112, higher scores indicating better quality-of-life. GDS scores ranging from 0–15, with higher scores indicating more severe depression. sMMSE scores range from 0–30, with a lower score indicating more cognitive impairment. CASP: for 19 item version scores range from 0–57 and for 12 item version scores range from 0–36, with higher scores indicating better quality-of-life. IPAQ-O social relations subscale scores range from 5–25, with higher score indicating more restriction in participation. PPOM total scores range from 0–64 and subscale scores range from 0–32, higher scores indicate better wellbeing.

## Discussion

This feasibility trial demonstrated that it is possible to conduct a definitive multi-center randomized controlled trial of the PRIDE intervention for people with dementia. A key strength of the study was our ability to recruit participants across varied sites in terms of local dementia service provision. With the exception of one site who adopted a wide net approach to screening, the retention rate from screening through to randomization was high, indicating that the screening procedures worked well and wide net approaches to recruitment are not efficient in terms of delivery of a trial such as ours. From a variety of recruitment methods, face to face invitations via the clinic or letters sent by the clinical sites were most successful. There was a high attendance rate for those receiving the intervention with non-attendance due to participants withdrawing from the trial, possibly due to losing momentum as sessions were spaced over 2 months. Across the sites we were able to train an adequate number of staff to act as facilitators, and local NHS Clinical Research Networks – CRNs – were very helpful and effective in sourcing research staff to recruit and collect data for this study.

Recruitment of study sites and participants is the key to the success of any trial.^27^ Following our field testing prior to this feasibility trial, we concluded that stringent site selection is important.^10^ Being clear on what is expected of the trial sites themselves enabled recruitment to be carried out more smoothly compared with our previous study.^3^ Requiring the sites to identify their own intervention facilitators was also a successful strategy. This was because in-house organization meant that time and resources could be better managed by the team themselves, who also had control over ensuring adequate staff were in place throughout the trial. This is vital in a trial that is resource heavy both at sites and within the research team, and the time required to keep the study on track due to the variability of services. Even with each site requiring to meet the same criteria, there were differences in site configuration and individual approaches, but this demonstrates a larger trial would be feasible even with variation. Maintaining good communication between the research team and the sites was required, especially for those with less research experience.

There was a high completion rate for all the outcome measures indicating that they were feasible to use in both arms of the study, and that the overall time taken and number of measures included was reasonable. Comparing the responsiveness to change of the candidate primary outcome measures in terms of wellbeing and independence, the CASP12 appeared to perform slightly better than the other options including the CASP 19, the IADL, and the DemQol. Further work is needed to estimate the minimal important change for these outcome measures as, due to the sample size in this study, confidence intervals for the estimates were wide. Preliminary evidence of the effectiveness of the intervention using these three outcome measures was, however, conflicting and did not signal an effect of the intervention on the CASP measure. Interpretation of this data should be treated with caution since this was not the main aim of this feasibility study. Taking into account the performance of the measures, a future trial could substitute the DemQol and the CASP19 for related shorter instruments such as the CASP-12 and other relevant measures arising from recent reviews on outcome measures for studies of this type.^27^,^28^

Given that people with dementia think that “in the moment” measurement is perhaps more reflective of their response to psychosocial interventions,^29^ there is scope to incorporate emerging digital Experience Sampling Methods (ESM)^30^ in a future definitive RCT of the PRIDE intervention.

The PRIDE intervention appears to be a useful and relevant way to try to improve independence and a range of activities for people with early stages of dementia. However, although it was popular and easy to use, at this stage we do not have a clear idea of the potential clinical benefits in practice. Based on this feasibility trial, we are now able to design a full scale protocol for a large multi-center trial of the PRIDE intervention compared to usual care, with some alterations and reductions to the range of outcome measures we examined.

It should be acknowledged, however, that the COVID-19 pandemic is likely to have a continued impact on care delivered in the community for people with dementia, but may also add limitations to what people are able to do in terms of activities outside the house. We suggest that the PRIDE intervention can be delivered with minimal contact in the home and is flexible in its tailoring to individual needs, preferences, and circumstances to be further tailored to individuals affected by the changes imposed through the current COVID-19 epidemic. Although only one participant used the online version, since the recent COVID-19 epidemic and its strategies for safety, many people have resorted to online resources in communication and gaining knowledge. Ongoing work from our research team on a refined PRIDE web-based solution will allow for tailoring of the PRIDE interventions including for those who have become further isolated and fearful during and beyond the epidemic.

## Conclusion

This study demonstrated that it is feasible to recruit to and carry out a multi-center trial of an individually tailored manualized PRIDE intervention in community-dwelling people who have mild dementia. The intervention was well received with a high completion rate and the outcome measures were completed to a high standard. Although measurable clinical benefit of the PRIDE intervention is not clear at present, a future large scale RCT has the potential to provide evidence of clinical and cost effectiveness. PRIDE is a relatively low-resourced 3-session intervention that is easy to deliver, and has scope to be scaled up across the health and social care services. The PRIDE web-application for tailoring towards those who have become seriously isolated during the current COVID-19 epidemic is an avenue for future study.

## Data Sharing Statement

The datasets generated during and/or analyzed during the current study will be available upon reasonable request from Nottingham Clinical Trials Unit.

Email: ctu@nottingham.ac.uk Telephone: +44 (0)115 8231600.
